# Translation, Cross-Cultural Adaptation, and Psychometric Analysis of the Indonesian Version of the Mayo Elbow Performance Score (MEPS-INA)

**DOI:** 10.7759/cureus.80106

**Published:** 2025-03-05

**Authors:** Iman W Aminata, Salman A Nizami

**Affiliations:** 1 Orthopaedics and Traumatology, Fatmawati General Hospital, Jakarta, IDN; 2 Faculty of Medicine, University of Indonesia, Jakarta, IDN

**Keywords:** elbow, mayo elbow performance score (meps), orthopedic, patient reported outcome measure, prom

## Abstract

Objective

This study aimed to translate and adapt the Mayo Elbow Performance Score (MEPS) into Indonesian (MEPS-INA) to evaluate its reliability and validity for Indonesian-speaking patients with elbow problems.

Methods

The translation process followed the forward and backward translation method as recommended by a previous study. The correlation between MEPS-INA and other measures (Medical Outcomes Study Short-Form of 12 items Health Survey (SF-12), Disabilities of the Arm, Shoulder and Hand (DASH), and Oxford Elbow Score (OES)) was analyzed to construct validity. Reliability testing value was using internal consistency coefficient, test-retest reliability, standard error of measurement (SEM), and minimal detectable change (MDC) values.

Results

The results confirmed significant correlations between the MEPS-INA with OES (0.74), DASH score (-0.61), SF-12 physical component score (PCS) (0.44), and SF-12 mental component summary (MCS) (0.23). Reliability testing showed good internal consistency (Cronbach's α of 0.93) with an intraclass correlation (ICC) of 0.93. MEPS-INA had low SEM, MDCindividual, and MDCgroup (1.74, 4.82, and 0.55, respectively).

Conclusion

MEPS-INA is a valid and reliable tool for assessing elbow function in Indonesian-speaking patients.

## Introduction

Over the past few years, the number of elbow procedures has increased due to improvements in surgical techniques, growing knowledge of elbow anatomy, and their accessibility. Currently, the most widely used outcome measure of elbow impairments in clinical trials worldwide is the Mayo Elbow Performance Score (MEPS) questionnaire, also referred to as the Mayo Elbow Performance Index (MEPI) [1-4].

Subjective patient-reported outcome measures (PROMs) offer useful information that has replaced the notion that physician-reported outcome measurements are the sole means of assessing treatment outcomes. The fundamental bias of clinician assessment and the way it tended to minimize patient perceptions of their results served as the basis for this paradigm shift [5]. Even though the MEPS is widely used worldwide, it is said to lack sufficient validation, and only a small number of translations and cross-cultural adaptations have been made.

The purposes of this study were to test the MEPS questionnaire for people with elbow issues by translating and adapting the English version into an Indonesian version (MEPS-INA). We hypothesized that the MEPS-INA would be reliable and valid for people who speak Indonesian.

## Materials and methods

Translation procedure

The forward and backward translation rules by Guillemin et al. are followed in the translation of MEPS-INA [6]. The translation process involves five key steps: initial translation, translation synthesis, back translation, committee review, and pretesting of the translated version. The final translated score was determined after all written reports were submitted and evaluated by an expert committee. Initially, two independent Indonesian translators (INA-1 and INA-2), fluent in English, completed the conceptual and literal translation of MEPS into Indonesian. A synthesized version (INA-12) was then developed by combining insights from these two translations. This synthesized version was back-translated into English by two qualified translators (ENG-1 and ENG-2) to identify any inconsistencies with the original English version. The final draft was pretested on 10 individuals as a preliminary trial. An expert committee, consisting of a methodologist, two experienced orthopedic surgeons specializing in shoulder and elbow, and a translator, reviewed the Indonesian translation. Based on the committee’s feedback, one of the authors (IWA) revised the questionnaire to create a pre-final draft. This draft was administered to patients with elbow issues, and one researcher (SAN) documented any challenges patients faced while completing the questionnaire. The expert committee analyzed these findings and used them to refine the questionnaire, resulting in the final MEPS-INA version (Table 1) [7].

**Table 1:** Indonesian Version of Mayo Elbow Performance Score (MEPS-INA) Translation of Original Mayo Elbow Performance Score (MEPS) into Indonesian language [7].

Variabel	Definisi	Poin
Nyeri (maksimal 45 poin)	Tidak ada nyeri	45
	Nyeri ringan	30
	Nyeri sedang	15
	Nyeri berat	0
Rentang gerak (maksimal 20 poin)	Rentang gerak > 100 derajat	20
	Rentang gerak diantara 50 – 100 derajat	15
	Rentang gerak < 50 derajat	5
Kestabilan sendi (maksimal 10 poin)	Stabil	10
	Tidak Stabil	5
	Sangat Tidak Stabil	0
Fungsional (maksimal 25 poin)	Mampu menyisir rambut	5
	Mampu makan sendiri	5
	Mampu melakukan aktivitas kebersihan pribadi	5
	Mampu memakai baju	5
	Mampu memakai sepatu	5
Hasil dapat dinilai dengan menggabungkan seluruh poin. Interpretasi nilai sebagai berikut; <60 poin – buruk; 60-74 poin – cukup; 75-89 poin – baik; 90-100 poin – sangat baik		

Patients and data collection

This investigation was approved by the Health Research Ethics Committee, National Institute of Health Research and Development (HREC-NIHRD), in accordance with Helsinki. A total of 80 consecutive patients who visited Fatmawati General Hospital between January 2024 and October 2024 were asked to participate in this study. All of the participants had elbow problems. Participants must be native speakers of Indonesian and have elbow issues verified by an experienced shoulder elbow orthopedic in order to be included in this study. Patients were given a first set of questionnaires (Section A) and after a week, the patients were asked to come back and complete Section B questionnaires.

Section A included the MEPS-INA along with three other questionnaires: the Medical Outcomes Study Short-Form of 12 items Health Survey (SF-12); the Disabilities of the Arm, Shoulder and Hand (DASH); and the Oxford Elbow Score (OES). Section B contained only the MEPS-INA and aimed to assess whether health status and elbow function remained consistent between the completion of Sections A and B. At the start of Section B, patients were asked a single question: "Has your elbow symptom changed since filling out the initial questionnaire?" This question was designed to determine if there were any changes in health status or elbow function between the two sections. Patients could respond with the following: (1) No; (2) Yes, the problem is improving; or (3) Yes, the problem is worsening. Only those who answered “No” regarding changes in their elbow symptoms were included in the test-retest analysis. During the waiting period, no interventions were carried out, and the patient was only given rehabilitation therapy exercises at home. Data from patients who completed section B more than one week later than section A are excluded from the analysis.

Instruments

The MEPS was a comprehensive evaluation method for elbow performance. In a single index, it integrates the subjective patient-based elements of pain and function with the clinically quantifiable components of mobility (ROM) and stability. The overall elbow performance was indicated by the total score, which was computed from the scores in each of the four dimensions and interpreted as follows: poor, fair, good, and excellent (Table 1). The MEPS had four domains consisting of pain, range of motion, joint stability, and elbow function. The scores range from 0 to 100. A lower score indicates a more severe elbow condition [7,8].

The SF-12, a generic health score, is utilized to create a health profile. The physical component summary score (PCS) and mental component summary score (MCS) are two of its domains. The PCS includes four key dimensions: physical functioning, role limitations due to physical health, bodily pain, and general health perceptions. Meanwhile, the MCS consists of vitality, social function, emotional role functioning, and mental health. Greater health status is indicated by higher scores of SF-12 (range from 0 to 100). The SF-12 version that was validated in the Indonesian language is utilized [9].

The 30-item DASH questionnaire assesses a patient's capacity to carry out specific upper extremity tasks. Patients can use a five-point Likert scale to score how difficult and disruptive their everyday lives are by answering this self-report questionnaire. The DASH score ranges from 0 to 100, where a higher score indicates a higher disability. A DASH is a valid and reliable questionnaire for upper extremity problems, and it has been translated into many other languages [10].

The OES is a validated questionnaire, specifically for elbow conditions, and has been translated into so many languages. Elbow function, pain, and social-psychological domains are the three categories into which the OES's 12 items are divided. There are five possible answers for each issue, and each one is rated on a scale of 0-4, where a lower score indicates more disability. The total score for the items is divided by the highest possible score, and the result is then multiplied by 100 to summarize the findings [11].

Validity

Since the MEPS has four distinct dimensions, namely, pain, range of motion, joint stability, and elbow function, we used a cross-sectional survey to assess the validity and reliability of both the four dimensions and the overall score. No manuscript or handling guidelines have been developed for MEPS translation. It is ultimately up to the investigator to carry out the implementation. To construct the validity of the MEPS-INA, we evaluated by establishing the correlation of MEPS-INA with questionnaires such as the SF-12, DASH score, and OES (Table 2).

**Table 2:** Hypothesis of the correlation coefficient of MEPS-INA with other questionnaires MEPS-INA (Indonesian version of Mayo Elbow Performance Score); SF-12 (Short-Form of 12 items Health Survey); PCS (Physical Component Score); MCS (Mental Component Score); DASH (Disabilities of the Arm, Shoulder, and Hand); OES (Oxford Elbow Score); ρ (Sperman rho coefficient)

Correlation	Hypothesized
MEPS-INA and SF-12 MCS	ρ < 0.25
MEPS-INA and SF-12 PCS	ρ > 0.25
MEPS-INA and DASH Score	ρ > 0.5
MEPS-INA and OES	ρ > 0.7

The MEPS-INA is assessed by analyzing its correlation with scores from the SF-12 (both PCS and MCS), the DASH score, and the OES. The SF-12 PCS is utilized to evaluate overall health and the level of pain experienced by patients. In contrast to PCS, the SF-12 MCS contains a questionnaire related to the patient's mental condition. Therefore, we have a hypothesis that PCS has a higher correlation than MCS since MEPS does not contain any information about the mental condition of the patient.

The DASH score contains questionnaires related to upper arm conditions, while the OES contains questionnaires related to specific elbow conditions. Since the OES is more elbow-specific than the DASH score, we have a hypothesis that the correlation between MEPS-INA and OES will be higher than MEPS-INA with the DASH score.

Floor and ceiling effects

The incidence of ceiling and floor effects was evaluated. If more than 15% of respondents have the lowest or highest possible score, then the results obtained are invalid or biased.

Reliability

Reliability refers to the ability to distinguish between individuals despite the presence of measurement errors. In line with the COSMIN (COnsensus-based Standards for the selection of health Measurement INstruments) guidelines, reliability was evaluated using internal consistency, test-retest reliability, and measurement error [12]. The degree of consistency in test measurements over multiple trials conducted under the same circumstances is estimated by internal consistency reliability. The consistency of measurement when administered to the same person more than once is known as test-retest reliability, and it shows how stable the scores are over time. The systematic error in a patient's score is being assessed by measurement error.

Statistical analysis

The characteristics of the study population and the scores from the questionnaires were analyzed using means and standard deviations (SDs) or frequencies and percentages. To assess construct validity, Spearman rho correlation coefficients were computed between the MEPS-INA scores and those of the other questionnaires. The Spearman rho values were interpreted by the Portney and Watkins classification [13]. Values <0.25, 0.25-0.5, 0.5-0.75, and >0.75 suggest little or no relationship, fair, moderate to good, and good to excellence, respectively. The internal consistency was established by Cronbach’s α. Values more than 0.70 are indicating good internal consistency [14].

The intraclass correlation coefficient (ICC) was used to assess the test-retest reliability of the MEPS-INA scores. ICC values below 0.5 indicate poor reliability, values between 0.5 and 0.75 suggest moderate reliability, values between 0.75 and 0.9 indicate good reliability, and values above 0.90 reflect excellent reliability [15]. Measurement error was evaluated by calculating the standard error of measurement (SEM) and the minimal detectable change (MDC). The SEM was derived by multiplying the pooled standard deviation (SD) by the square root of (1 − r), where (r) represents the ICC of the test and retest scores. The MDC at the individual level (MDCindividual) was computed using the formula 1.96 × SEM × √2, while the MDC at the group level (MDCgroup) was determined by dividing MDCindividual by √n, where (n) is the total number of samples [14].

Bland-Altman’s plots were utilized to assess absolute reliability; no systematic bias is present when 0 is in the 95% confidence interval (CI) of the mean difference between the first and second administration of the MEPS-INA. The 95% limits of agreement (LOA) were determined with the formula mean difference ± 1.96 × SDdiff, where SDdiff is the SD of the mean difference between the first and second administration of the MEPS-INA [16]. Proportional bias was examined using linear regression. All statistical analyses were performed using Statistical Product and Service Solutions (SPSS, version 25; IBM SPSS Statistics for Windows, Armonk, NY).

## Results

Patient characteristics

There was a total of 80 patients who had filled out the Section A questionnaire. However, there were three patients who did not return to fill out the Section B questionnaire and two patients who felt that their elbow condition was much better (Table 3). Therefore, the total population that could be analyzed in this study was 75 patients (94% of total participants). The age range of the patients who completed the questionnaire was 17-72 years old. The most common cases encountered were fractures or malunions. The second MEPS-INA measurement score was slightly better than the MEPS-INA measurement results one week earlier (Table 4).

**Table 3:** Demographic data of patients (n=75)

Characteristics	Mean ± SD or n (%)
Age (years)	36.4 ± 15.3
Sex	
Male	41 (54.7)
Female	34 (45.3)
Dexterity	
Right-Handed	64 (85.4)
Left-Handed	11 (14.6)
Affected Elbow	
Right	33 (44)
Left	42 (66)
Diagnosis	
Stiff Elbow	16 (21.3)
Fracture/Malunion	27 (36)
Dislocation	17 (22.6)
Infection/Osteomyelitis	8 (10.6)
Varus Deformity	1 (1.3)
Arthritis	2 (2.6)
Instability	4 (5.3)

**Table 4:** Outcome of the patient-reported outcome measures (PROM) questionnaire MEPS-INA (Indonesian version of Mayo Elbow Performance Score); SF-12 (12-Item Short-Form Health Survey); PCS (Physical Component Score); MCS (Mental Component Score); DASH (Disabilities of the Arm, Shoulder, and Hand); OES (Oxford Elbow Score)

Characteristic	Mean ± SD
Section A	
MEPS-INA	67.7 ± 12.8
SF-12 PCS	38.8 ± 6
SF-12 MCS	47.9 ± 4.4
DASH Score	42.1 ± 12.3
OES	66.2 ± 12.1
Section B	
MEPS-INA	69 ± 13.7

Validity

All of the predetermined hypotheses regarding the strength of the correlations between the MEPS-INA and the PCS, MCS, DASH score, and OES were verified. As anticipated, the MEPS-INA was strongly correlated with the OES other than the SF-12 and DASH score (Table 5). There were no floor or ceiling effects from the investigation.

**Table 5:** The Spearman ρ correlation coefficient of MEPS-INA MEPS-INA (Indonesian version of Mayo Elbow Performance Score); SF-12 (12-Item Short-Form Health Survey); PCS (Physical Component Score); MCS (Mental Component Score); DASH (Disabilities of the Arm, Shoulder, and Hand); OES (Oxford Elbow Score); *significant

Outcome	Correlation	p-value
MEPS-INA and SF-12 PCS	0.44	p < 0.001*
MEPS-INA and SF-12 MCS	0.23	p < 0.001*
MEPS-INA and DASH	-0.6	p < 0.05*
MEPS-INA and OES	0.74	p < 0.001*

Reliability

Cronbach's α was 0.93, demonstrating strong internal consistency. The ICC value was 0.93 (p < 0.001), with a 95% confidence interval ranging from 0.89 to 0.95. The SEM was calculated as 1.74, while the minimal detectable change for individuals (MDCindividual) was 4.82, and for groups (MDCgroup), it was 0.55. The Bland-Altman approach revealed a mean difference between the test and retest MEPS-INA score of 1.26 (95% CI, -2.78 to 2.47, 95% LOA, -14.16 to 11.63). Linear regression was employed to evaluate the proportional bias with the mean coefficient was 0.07 (p = 0.21). Therefore, no proportional bias in this data selection.

## Discussion

The purposes of this research were to translate and cross-culturally adjust the original MEPS into Indonesian. This research discovered more about MEPS-INA validity and reliability. The MEPS-INA has been established as both valid and reliable for use among Indonesian-speaking populations.

Its constructed validity is considered strong, as all predefined hypotheses were supported. Consistent with expectations, the MEPS-INA demonstrated a high correlation with the OES (ρ = 0.74). This was comparable with previous studies in Denmark and Greek versions that showed a good correlation between the MEPS and OES (ρ = 0.80 and 0.71, respectively) [17,18]. MEPS-INA showed a good correlation with the DASH score (ρ = 0.61). Our study was in line with a previous correlation study in MEPS Greek with a DASH score (ρ = 0.64) [18]. As expected, the MEPS-INA exhibited a weak correlation with the MCS (ρ = 0.23). In contrast, it showed a stronger correlation with the PCS domains (ρ = 0.44), aligning with our hypothesis. A similar pattern was observed in the MEPS Turkey, which also demonstrated comparable correlations with the PCS and MCS of the SF questionnaire [19]. The MEPS-INA was primarily developed to evaluate pain and physical function rather than social or emotional factors. As a result, its correlations with the PCS were significantly stronger than those with the MCS.

This study found no floor or ceiling effects, highlighting the MEPS-INA's ability to effectively differentiate among patients with elbow issues. Similar findings were observed in other language versions, such as the Turkish adaptation, further supporting the absence of such effects [19]. These results suggest that the MEPS-INA has strong content validity. Additionally, the internal consistency of the MEPS-INA is excellent, as indicated by Cronbach's α of 0.93. This aligns with previous studies evaluating MEPS in patients with elbow dysfunction [20].

The MEPS-INA demonstrated strong test-retest reliability, with an ICC of 0.93, which is similar to the Turkish version's ICC of 0.89 [19]. Following the COSMIN guidelines, the time gap between the test and retest was carefully chosen to minimize recall bias while ensuring the measured values remained relevant. This approach ensured that the data being assessed stayed consistent and unchanged. Based on this result, the period of one-week intervals is possibly adequate to perform test-retest reliability among patients with elbow problems.

MEPS-INA had low SEM, MDCindividual, and MDCgroup values (1.74, 4.82, and 0.55, respectively). For group-level comparisons, MEPS-INA showed sufficient capability as change can be detected with small values. To detect a statistically significant change in MEPS-INA scores at the group level, the difference must exceed 1.74, as only values greater than the SEM can reliably be distinguished from measurement error. For individual patients, the difference between two measurements must surpass the MDCindividual value and the SEM to confirm that an actual change has occurred, rather than being attributed to measurement error. MEPS-INA was a significant tool for tracking individual patients over time, especially since MDCindividual is relatively small (4.82).

Limitation

The study has certain limitations. First, some patients did not appear for the second meeting to fill the section B questionnaire. Second, because of the limited availability of the official Indonesian version of the valid questionnaire, we used the self-translated version of the OES and DASH score. No information is available concerning the psychometric properties of the Indonesian version of the OES and DASH score. However, we performed a translation process following the international guidelines as suggested by Guilleminet al. [6].

## Conclusions

The MEPS-INA is particularly effective for the Indonesian population with elbow cases. The results of this study indicate that MEPS-INA is an acceptable and credible tool for evaluating elbows in the Indonesian population.
